# Aberrant Hedgehog Signaling and Clinical Outcome in Osteosarcoma

**DOI:** 10.1155/2014/261804

**Published:** 2014-03-30

**Authors:** Winnie W. Lo, Dushanthi Pinnaduwage, Nalan Gokgoz, Jay S. Wunder, Irene L. Andrulis

**Affiliations:** ^1^Department of Molecular Genetics, University of Toronto, ON, Canada M5S 1A8; ^2^Lunenfeld-Tanenbaum Research Institute, Mount Sinai Hospital, 600 University Avenue, Toronto, ON, Canada M5G 1X5; ^3^University Musculoskeletal Oncology Unit, Mount Sinai Hospital, Toronto, ON, Canada M5G 1X5; ^4^Department of Surgery, University of Toronto, ON, Canada M5G 1L5

## Abstract

Despite the importance of Hedgehog signaling in bone development, the relationship between Hedgehog pathway expression and osteosarcoma clinical characteristics and outcome has not been investigated. In this study of 43 high-grade human osteosarcoma samples, we detected high expression levels of the Hedgehog ligand gene, *IHH*, and target genes, *PTCH1* and *GLI1*, in most samples. Further analysis in tumors of patients with localized disease at diagnosis identified coexpression of *IHH* and *PTCH1* exclusively in large tumors. Higher levels of *IHH* were observed more frequently in males and patients with higher levels of *GLI1* were more responsive to chemotherapy. Subgroup analysis by tumor size and *IHH* expression indicated that the well-known association between survival and tumor size was further refined when *IHH* levels were taken into consideration.

## 1. Introduction

Osteosarcoma is the most common primary bone cancer and typically affects children and young adults. Although osteosarcoma is relatively rare, the disease afflicts individuals in the prime of their productive life and the clinical behaviour is highly aggressive [1]. Almost all osteosarcomas are high grade and have a poor prognosis, with 10–20% of patients having detectable metastases to the lungs at diagnosis. With the combination of surgery and chemotherapy, 50–60% of patients with a localized osteosarcoma will achieve long term disease-free survival, while the long term survival for patients with metastasis at diagnosis is only 20–30% [2–4].

The Hedgehog (Hh) signaling pathway is crucial for the regulation of proliferation and differentiation during embryonic development (for reviews, see [5, 6]), and its complexity is reflected from the involvement of multiple regulatory proteins at different cellular levels. In the absence of Hh ligands, patched homologue (PTCH1), a twelve-transmembrane protein, inhibits the localization of smoothened homologue (SMO), a seven-transmembrane protein, to cilia, thus preventing the activation of downstream signaling. Binding of the Hedgehog ligand to PTCH1 prevents PTCH1-mediated inhibition of SMO, thereby allowing SMO to localize to cilia and promote downstream activation of transcription factors encoded by the GLI zinc-finger family. In vertebrates, there are three Hedgehog-family ligands, sonic (SHH), Indian (IHH), and desert (DHH), and three GLI proteins. GLI1 and GLI2 act mainly as transcriptional activators, while GLI3 functions mainly as a repressor. Expression of GLI activators results in the induction of Hh target genes, including* GLI1* and* PTCH1*, that participate in positive and negative feedback mechanisms.

The Hh pathway is dormant in most adult tissues but becomes aberrantly activated in many cancers in a ligand-dependent or ligand-independent manner [6–12]. Both mechanisms are accompanied by overexpression of downstream Hh pathway targets, such as* PTCH1* and* GLI1*. Ligand-dependent Hh activation is caused by overproduction of the Hh ligand. Inactivating mutations of* PTCH1* are the most common causes of ligand-independent activation and are frequently found in basal cell carcinomas and medulloblastomas [13–16].

The origin of osteosarcoma is unknown, although these tumors tend to form in areas of rapid bone growth or turnover, such as in the long bones of developing adolescents. The long bones of the limbs and ribs develop by a process known as endochondral ossification. The Hedgehog pathway, more specifically the Indian Hedgehog (Ihh) ligand, regulates endochondral ossification by coordinating chondrocyte proliferation and differentiation and osteoblast differentiation [17]. Bone formation has been demonstrated to be reduced in* Ihh* ^−/−^ mice [18]. In addition, the IHH pathway has been found to be constitutively active in chondrosarcoma [19–22]; however, the role of Hedgehog signaling in osteosarcoma has not been investigated extensively. We previously identified Hedgehog dysregulation and signaling crosstalk between the tumor and the stroma in osteosarcoma cell lines and patient-derived xenograft models and demonstrated specific blockage of signaling exerted by Hedgehog inhibitors [23]. In the current study, we determined the levels of expression of Hedgehog pathway components (*IHH*,* SMO*,* PTCH1*, and* GLI1*) in primary human osteosarcoma specimens and examined the association with clinical characteristics and patient outcome.

## 2. Materials and Methods

### 2.1. Tumor Specimen

Tumors from forty-three patients with high-grade osteosarcoma of the extremity were evaluated in this study. Eleven were obtained from patients who had metastases at diagnosis, and the remaining thirty-two were from patients who had no metastasis at diagnosis. Of these thirty-two samples, twenty were from patients who remained free of systemic disease, and twelve were from those who developed metastasis after diagnosis. Patients were treated at 1 of 5 tertiary care medical institutions: Mount Sinai Hospital, Toronto, Canada; Vancouver Hospital and Health Sciences Center, Vancouver, Canada; Royal Orthopaedic Hospital, Birmingham, UK; Memorial Sloan Kettering Cancer Center, New York, NY, USA; and Mayo Clinic, Rochester, MN. Patients were seen in regular follow-up for at least 4 years from the time of diagnosis or until systemic recurrence, except for patients who presented with metastases at the time of diagnosis.

Patients were selected from a larger tumor bank cohort on the basis of availability of tumor material. Each eligible patient provided a signed consent form before study entry, as approved by each participating institution's Research Ethics Board. Tumor specimens were obtained at the time of surgical biopsy and were chosen by a pathologist with the aid of frozen histological analysis to ensure the presence of viable tumor without normal tissue contamination. Tumor samples were collected immediately after surgery, snap-frozen in liquid nitrogen, and stored at −70°C.

### 2.2. Differentiation of Human Osteoblastic Cells

Human bone marrow from two cancer-free individuals was obtained directly from surgical specimens. Bone marrow specimens were washed with Minimum Essential Medium Alpha with 300 U of Penicillin-Streptomycin for 15 to 20 minutes and repeated 2 times with fresh media with antibiotics. To differentiate cells of the osteoblastic lineage, tissues were cut into small pieces, placed in 10 cm plates, and cultured in fully supplemented media (*α*-MEM with 15% FBS, 100 U Penicillin-Streptomycin, 50 *μ*g ascorbic acid/mL, 10 mM Na *β*-glycerophosphate, and 10^-8 ^M dexamethasone) at 37°C undisturbed for 3 days. Fresh media were replaced every 3 to 4 days with regular monitoring under the microscope. Tissues were removed once spindle-shaped cells became visible for 1 week or more. Cells were cultured until confluent, at which time the cells were collected for RNA and DNA extraction or trypsinized and replated in 96-well plates. Differentiated cells were fixed and evaluated for the presence of alkaline phosphatase, using Naphthol AS phosphate (Sigma) coupled with Fast Blue BB Salt (Sigma), and for mineralization, using 0.2% Alizarin Red S solution (Sigma).

### 2.3. Quantitative Assessment of* IHH*,* SMO*,* PTCH1*, and* GLI1* mRNA Expression by Real-Time RT-PCR

Frozen tumor samples were crushed in a Brinkman Retch crusher. Total RNA was extracted using TRIzol (Invitrogen). Total RNA was reverse transcribed to cDNA using M-MLV Reverse Transcriptase (Invitrogen). Gene-specific TaqMan Assay-on-Demand (Applied Biosystems) was used to quantify the transcript levels of* IHH, SMO, GLI1, *and* PTCH1* in the samples. The absolute standard curve method was used to determine the levels of expression by relating the PCR signal to a standard curve that was first constructed from RNA of known concentration. This curve was then used as a reference standard for extrapolating quantitative information for mRNA targets of unknown concentrations. cDNA from colon cancer cell lines SW1417 and RW948 was used as reference standards for* IHH* and* PTCH1*, respectively, because of the known high levels of expression found in these cells. cDNA from a pool of 11 tumor cell lines was used as a reference standard for* SMO* and* GLI1*. Asparagine synthetase (*AS*) was used as an endogenous control for expression normalization of* IHH*, while glyceraldehyde-3-phosphate dehydrogenase (*GAPDH*) was used for* SMO*,* GLI1*, and* PTCH1*. The housekeeping genes* AS* and* GAPDH* were chosen based on their similarity in the dynamic quantification range to the genes of interest.

### 2.4. Data Analysis and Statistical Tests

The expression values of* IHH*,* SMO, PTCH1*, and* GLI1* were log2 transformed to achieve approximate normality of the error distribution. Histograms showed that the log2 transformed expression data were still skewed to the left, with the exception of* GLI1* data. Therefore, the median was taken as a measure of center, and samples were assigned to the “High” or “Low” expression group, using the median as a cutoff. Descriptive baseline analyses by Chi-square test or Fisher's exact test or* t*-test compared frequency distribution of selected clinical and demographic variables among groups defined by expression status (high versus low expression, using the median as cutoff). Categoric or continuous coding schemes for the variables were selected before the analysis, on the basis of previous study or clinical convention. The selected clinical and demographic variables included were tumor size (>9 cm versus ≤9 cm), chemotherapy-induced necrosis (>90% versus ≤90%), gender (female versus male), and age at diagnosis (continuous). Univariate disease-free survival (metastasis-free, DFS) analysis according to subgroups of expression status was assessed by the Log-Rank test with Kaplan-Meier survival curves and by the Cox Proportional Hazards model. The Cox Proportional Hazards model was used for multivariate DFS analysis to assess the contribution of each gene in addition to traditional prognostic factors. Prognostic factors included in the analysis were clinical and demographic variables mentioned above. Relative risks (RR) for each gene and each factor were estimated by the hazard ratio in the Cox Proportional Hazards model [24, 25]. To further assess the contribution of each gene status in combination with tumor size (dichotomous), univariate and multivariate survival analyses were repeated considering the four groups of gene status and tumor size (high/>9 cm, low/>9 cm, high/≤9 cm, and low/≤9 cm) as the main variable in each model. Firth's correction was applied to the Cox modeling with sparse data [26].

All tests were two sided. Disease-free survival (DFS) was taken as the time between diagnosis and the confirmation of metastasis. Patient status on October 26, 2012, determined DFS times and censoring status using clinical follow-up data. We observed that 15 patients developed metastases out of 32. There were no lost to follow-ups. Excluding the patients with metastases, the minimum follow-up time was 60 months and the median follow-up time was 108 months. Patients with disease-free status were censored at the last follow-up date. A test with a *P* value <0.05 was considered statistically significant. All statistical analyses were performed using SAS 9.2 software (SAS Inc., Cary, NC, USA) with *P* values unadjusted for multiple testing and Kaplan-Meier plots generated using R statistical software version 2.3.0 (http://www.r-project.org/).

## 3. Results

### 3.1. High Levels of Expression of Indian Hedgehog Genes in Osteosarcoma Samples

**Figure 1:**
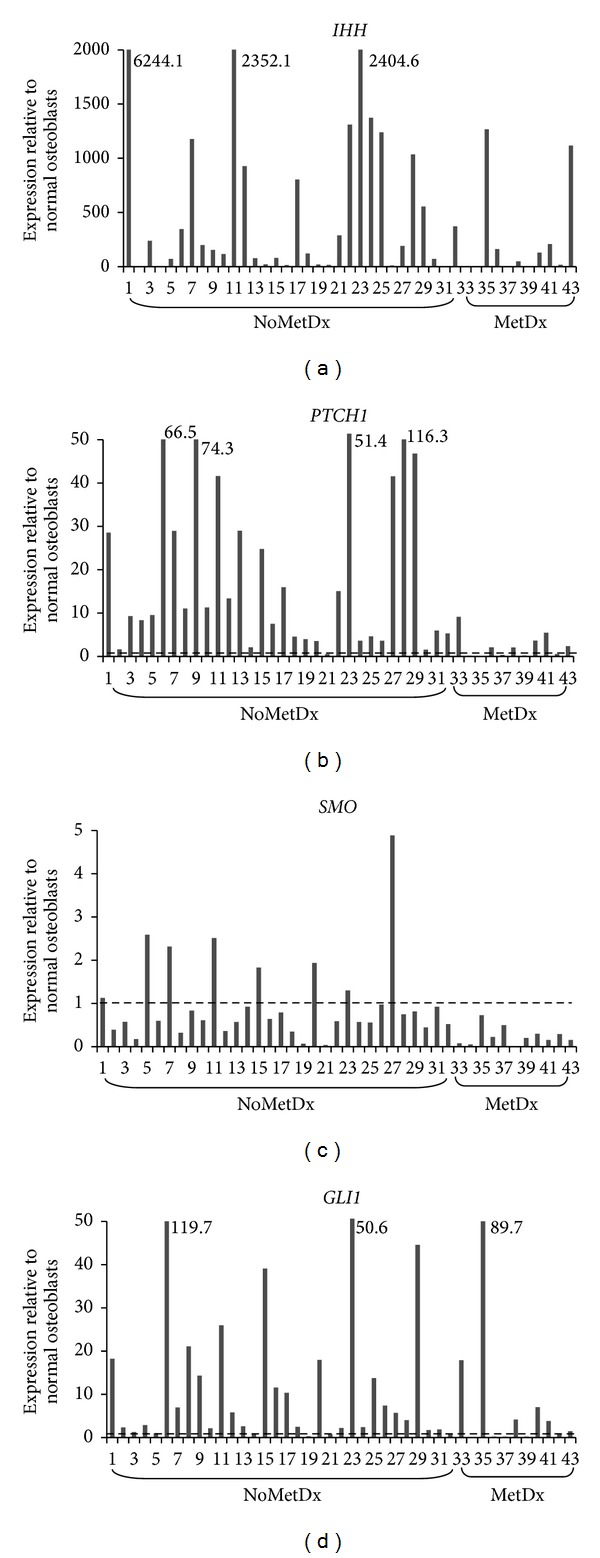
Expression levels of* IHH*,* PTCH1*,* SMO, *and* GLI1* in 43 osteosarcoma tumors relative to normal osteoblasts. Expression levels in tumors were normalized to the average level of 2 normal osteoblast samples and presented as fold change. Fold changes greater than the *y*-axis are labelled next to the representing bars (NoMetDx = no metastasis at diagnosis; MetDx = metastasis at diagnosis).

Since there are currently no specific antibodies for endogenous human Hedgehog pathway genes that work reliably in immunohistochemistry assays [27], the transcript levels of* IHH*,* SMO*,* PTCH1*, and* GLI1 *were determined using real-time RT-PCR in 43 human primary high-grade osteosarcoma samples and normalized to the levels detected in normal osteoblasts (Figure 1). Variable levels of* IHH*,* SMO*,* PTCH1*, and* GLI1* expression were observed in the osteosarcoma samples. The expression levels of* IHH *were low in the normal osteoblasts and most tumors exhibited higher* IHH* levels relative to the normal osteoblasts. Likewise, the majority of the samples exhibited higher than normal levels of* PTCH1 *and* GLI1*, with some exhibiting very high levels of expression. With the exception of a few specimens,* SMO* was not highly expressed in the tumors.

### 3.2. Coexpression of Indian Hedgehog Pathway Genes in Tumors from Subjects Presenting without Metastasis at Diagnosis

Thirty to forty percent of osteosarcoma patients who have localized disease at diagnosis will eventually develop metastasis. To determine whether expression of the Hh pathway genes is associated with patient clinical characteristics and outcome, expression was further analyzed in the subgroup (*n* = 32) presenting without metastasis at diagnosis (NoMetDx). In this subgroup, the mean age was 26.5 years (SD = 17.3, minimum = 7, and maximum = 73); 68.8% were male; 46.9% had small tumors; 46.9% had large tumors; 6.2% (2 tumors) had no tumor size data; 50% of tumors had low necrosis; 28.1% had high necrosis; 21.9% (7 tumors) had no necrosis data. Furthermore, as shown in Table 1, with the exception of the* IHH* and* SMO* pair, the levels of* IHH*,* PTCH1*,* SMO, *and* GLI1 *are positively correlated with each other in tumors from patients presenting without metastasis at diagnosis. Interestingly, a strong coexpression of* IHH* with* PTCH1 *was observed only in the subgroup of large tumors (*n* = 15), whereas expression of* PTCH1*,* SMO*, and* GLI1 *was coexpressed in small tumors (*n* = 15) (Table 1).

### 3.3. Associations between Levels of Hedgehog Pathway Components with Clinical Characteristics in Tumors from Subjects Presenting without Metastasis at Diagnosis

Gene expression levels were not related to clinical characteristics (data not shown) with the exception of an association of* IHH* expression with gender and* GLI1* expression with chemotherapy-induced necrosis. Higher levels of* IHH* were observed more frequently in males than in females (72.7% versus 20.0%; *P* = 0.0084). Patients with higher levels of* GLI1* were more responsive to chemotherapy as demonstrated by higher percentage of chemotherapy-induced necrosis compared to patients with lower* GLI1* levels (53.3% versus 10.0%; *P* = 0.0405).

### 3.4. Effects of* IHH *and* PTCH1 *Levels on Survival

**Figure 2:**
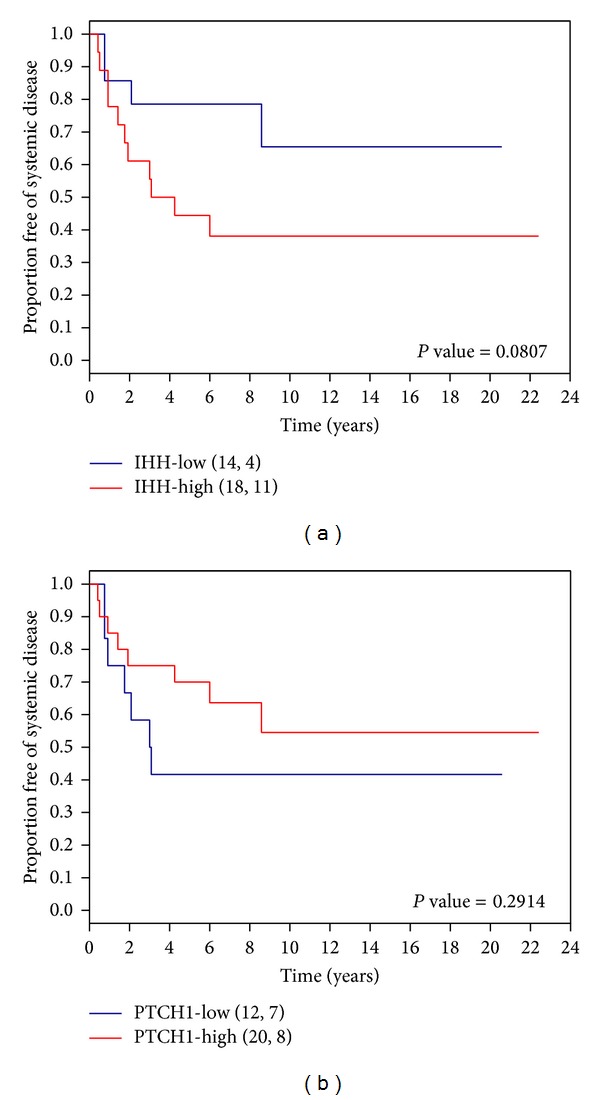
Kaplan-Meier survival curves of systemic disease-free survival for patients without metastasis at diagnosis with high and low levels of (a)* IHH* and (b)* PTCH1* gene expression. *P* value = Log-Rank *P* value.

Log-Rank tests suggested a borderline significant survival difference for high versus low expression of* IHH* (*P* value = 0.0807). A trend toward a worse disease-free survival was observed for patients in the high* IHH* expression group compared to patients in the low* IHH *expression group from the univariate Cox Proportional Hazards model (*n* = 32) (*P* = 0.0948, hazard ratio (HR) = 2.66; confidence interval (CI): (0.84, 8.40)) (Table 2; Figure 2(a)). In contrast, a trend towards better survival was found in patients in the high* PTCH1* group (*P* = 0.2927; HR = 1.73; CI: (0.62, 4.79)) (Figure 2(b)). No survival differences were identified in the high and low expression groups of* GLI1 *and* SMO *(data not shown).

In the multivariate Cox Proportional Hazards model (*n* = 23) (Table 2), to assess whether tumor size in combination with the expression of* IHH* correlates with poor survival, borderline significance (*P* = 0.0970; HR = 6.4; CI: (0.7, 57.9)) was achieved for high* IHH* expression group. No statistically significant difference for* PTCH1 *was detected in the multivariate model (data not shown).

### 3.5. Ability of* IHH* Levels to Better Predict Outcome Groups in Patients with Different Tumor Sizes

**Figure 3:**
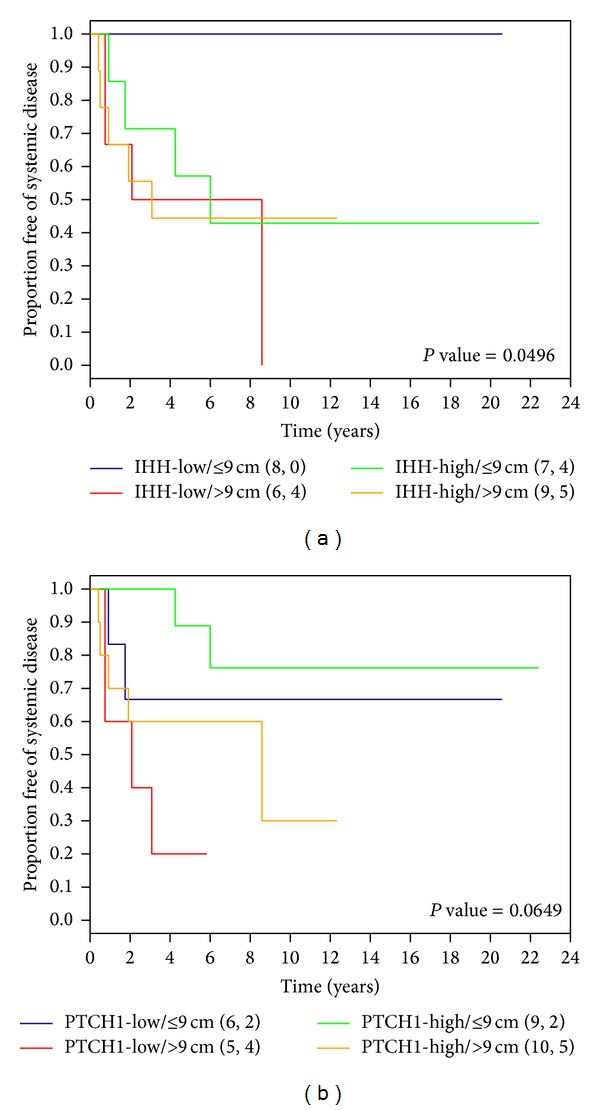
Kaplan-Meier survival curves of systemic disease-free survival for patients without metastasis at diagnosis in combination with tumor size and (a)* IHH* or (b)* PTCH1* expression status. *P* value = Log-Rank *P* value.

Disease-free survival was compared among patients with tumors exhibiting different combinations of size (>9 cm or ≤9 cm) and gene expression status (high or low). Interestingly, we found that the group with both low* IHH* expression and smaller tumors remained disease-free and thus had the best disease-free survival compared to the other three groups (Log-Rank *P* value = 0.0496 (for 4-group comparison)) (Figure 3(a)). Moreover, in the small tumor subgroup, patients with low* IHH* expression had better disease-free survival than those with high* IHH *expression (*P* value = 0.0163; HR = 3.19; CI: (0.58, 17.44)). These results suggest that* IHH* levels can better predict outcome in patients with small tumors. We also found that the group with both low* PTCH1* expression and larger tumors tended to have poorer disease-free survival compared to the other three groups (Log-Rank *P* value = 0.0649 (for 4-group comparison)) (Figure 3(b)).

Furthermore, a multivariate model for* IHH* in combination with size (Table 3) shows that* IHH* in combination with size can identify a higher risk group than* IHH* or size alone. Patients in the large tumor and high* IHH* group are at much higher risk compared to those in the smaller tumor and low* IHH* group (low risk group) (*P* = 0.0378; HR = 52.30; CI: 3.27, 10906.34). We also noticed that when compared to the low risk group, patients in the large tumor and low* IHH* group are at a trend of lower risk than those in the large tumor and high* IHH* group (HR: 17.62 versus 52.3) (Table 3). Furthermore, patients in the small tumor and high* IHH* group are at a trend of higher risk than those in the small tumor and low* IHH* group (low risk group) (*P* = 0.2831; HR = 6.31; CI: 0.47, 934.10).

## 4. Discussion

While previous expression studies have suggested the involvement of dysregulated Hedgehog signaling in osteosarcoma, most of them were carried out in osteosarcoma cell lines with the inclusion of limited numbers of clinical samples [28–32]. The transcript levels of Hedgehog pathway genes have been associated with outcome in many cancer types [33, 34], but this is the first report to show the relationship between Hedgehog pathway gene expression and osteosarcoma outcome.

In this study of 43 osteosarcoma samples, we detected high expression levels of the Hedgehog ligand gene,* IHH, *and IHH target genes,* PTCH1* and* GLI1*, in most osteosarcoma samples. We found that tumors from patients presenting without metastasis at diagnosis exhibited pairwise coexpression of* IHH*,* PTCH1*,* SMO, *and* GLI1 *with the exception of the pair of* IHH* and* SMO*. In addition, a correlation of expression of the ligand gene,* IHH*, and the target gene,* PTCH1*, found exclusively in large tumors, is indicative of ligand-dependent activation. In contrast, levels of* SMO, PTCH1*, and* GLI1* were found to be positively correlated with each other in small tumors, suggesting ligand-independent activation. These data suggest that both ligand-dependent and ligand-independent mechanisms may lead to Hedgehog activation in osteosarcoma but having ligand-dependent activation due to high levels of* IHH* may lead to larger tumor size, which is a well-known prognostic factor for osteosarcoma. Indeed, we found that patients with higher levels of* IHH* tended to exhibit a worse outcome compared to those with lower levels of expression (HR = 2.66; CI: (0.84, 8.40)). Interestingly, even though the levels of* IHH* and* PTCH1 *are coexpressed, patients with higher levels of* PTCH1* (HR = 1.73; CI: (0.62, 4.79)) tended to have a better outcome.

In this exploratory study, we did not detect statistically significant differences with our limited sample size; however, HR values as large as 8.4 and 4.8 cannot be ignored. Furthermore, the opposite effects of high levels of* IHH* and* PTCH1* on survival are in line with the biological roles of IHH and PTCH1 in the pathway. The PTCH1 receptor negatively regulates signaling in the absence of Hh ligands by preventing the SMO receptor from activating downstream signaling. The Hh ligands positively regulate signaling by binding to PTCH1 and relieving its inhibition of SMO, thereby allowing SMO to induce downstream signaling.

Nonetheless, even with our limited sample size, we found that the well-known association between survival and tumor size was further refined when* IHH* levels were taken into consideration. Patients with lower* IHH *expression and small tumors remained disease-free and had better survival compared to patients with higher* IHH *expression and small tumors which demonstrated similar survival to those with large tumors. Similarly in the large tumor group, patients with lower* IHH* tumors had better survival than those with higher* IHH *expression. Since the Hedgehog pathway controls the proliferation of chondrocytes during bone development [17], an interaction between IHH and proliferation, which presumably contribute to tumor size, likely exists. However, the available sample size limits the precision of the results obtained (see wide confidence intervals in Table 3); therefore, it will be important to replicate the results in a larger study.

Our descriptive analyses demonstrated that higher levels of* IHH *were more frequently observed in males than in females. The incidence of osteosarcoma is more frequent in males than females [35], which is also found in our cohort (68.8% male versus 31.2% female). In addition, in our cohorts, males tend to have larger tumors than females (80% versus 20%; *P* = 0.12). High levels of* IHH* found in males may suggest that ligand-dependent Hedgehog activation may contribute to abnormal cell proliferation and thus larger tumors and higher incidence of osteosarcoma in males. A positive correlation was identified between* GLI1 *expression and chemotherapy-induced necrosis. This could suggest that although GLI1 is a positive regulator of the pathway, tumors with higher levels of* GLI1* may be more responsive to therapy. Future studies that include larger number of osteosarcoma cases will be required to further examine the relationship between the Hedgehog pathway genes and clinical characteristics and outcome.

The observation that* IHH* expression is predictive of outcome in combination with tumor size suggests that* IHH* expression may be used to improve the prognostic value of tumor size and possibly improve the stratification of patients prior to starting chemotherapy. For instance, patients determined to be at lower risk of developing metastasis due to both small tumor size and low* IHH* expression may require less prolonged or less intensive chemotherapy treatment to prevent development of metastatic disease. In addition, with the development of inhibitors that target aberrant Hh signaling, it will be important to determine whether our findings can be replicated in larger study cohorts to identify patients with tumors exhibiting constitutive ligand-dependent signaling that may also be responsive to Hedgehog inhibitors.

## Figures and Tables

**Table 1:** Pairwise Pearson correlation coefficients between log-transformed *IHH*, *PTCH*1, *GLI*1, and *SMO* expression in osteosarcoma samples and *P* value for test of the null hypothesis that correlation is zero.

	NoMetDx (*n* = 32)				Large tumors (*n* = 15)				Small tumors (*n* = 15)			
	log⁡IHH	log⁡PTCH1	log⁡SMO	log⁡GLI1	log⁡IHH	log⁡PTCH1	log⁡SMO	log⁡GLI1	log⁡IHH	log⁡PTCH1	log⁡SMO	log⁡GLI1
log⁡IHH	1.00	**0.44 **(**P** = 0.011)	0.20 (*P* = 0.27)	**0.34 **(**P** = 0.056)	1.00	**0.56 **(**P** = 0.029)	0.05 (*P* = 0.86)	0.39 (*P* = 0.15)	1.00	0.37 (*P* = 0.18)	0.28 (*P* = 0.31)	0.34 (*P* = 0.21)

log⁡PTCH1		1.00	**0.52 ** (**P** = 0.002)	**0.61 **(**P** = 0.0002)		1.00	0.43 (*P* = 0.11)	0.44 (*P* = 0.10)		1.00	**0.53 **(**P** = 0.04)	**0.70 **(**P** = 0.004)

log⁡SMO			1.00	**0.45 **(**P** = 0.009)			1.00	0.24 (*P* = 0.40)			1.00	**0.56 **(**P** = 0.03)

log⁡GLI1				1.00				1.00				1.00

Bold text indicates statistically significant positive correlations.

Two patients have missing tumor size information.

**Table 2:** Results of DFS analysis by Cox Proportional Hazards model in no metastasis at diagnosis group (*n* = 32) for IHH.

Prognostic factor	Univariate				Multivariate*			
	HR	95% CI		*P* value	HR	95% CI		*P* value
IHH								
High versus low	2.66	0.84	8.40	0.0948	6.43	0.71	57.97	0.0970
Size								
>9 cm versus ≤9 cm	3.52	1.06	11.67	0.0394	22.81	2.22	234.02	0.0085
Necrosis								
>90% versus ≤90%	0.71	0.21	2.35	0.5716	0.53	0.13	2.14	0.3733
Gender								
Male versus female	1.39	0.44	4.36	0.5795	0.22	0.02	2.25	0.1998
Age (years)	1.02	0.99	1.05	0.1676	1.03	0.99	1.07	0.0965

*Firth correction to handle small sample sizes.

**Table 3:** Results of DFS analysis by Cox Proportional Hazards model in no metastasis at diagnosis group (*n* = 32) for IHH in combination with size.

Prognostic factor	Univariate				Multivariate*			
	HR	95% CI		*P* value	HR	95% CI		*P* value
IHH/size								
IHH+/≤9 cm versus IHH−/≤9 cm	12.78	1.36	1694.88	0.1128*	6.31	0.47	934.10	0.2831
IHH+/>9 cm versus IHH−/≤9 cm	17.10	1.91	2253.96	0.0754*	52.30	3.27	10906.34	0.0378
IHH−/>9 cm versus IHH−/≤9 cm	20.48	2.14	2727.27	0.0611*	17.62	1.48	2614.55	0.0907
Necrosis								
>90% versus ≤90%	0.71	0.21	2.35	0.5716	0.62	0.16	2.39	0.5027
Gender								
Male versus female	1.39	0.44	4.36	0.5795	0.33	0.03	2.10	0.2700
Age (years)	1.02	0.99	1.05	0.1676	1.03	0.99	1.07	0.2034

*Firth correction to handle small sample sizes.

IHH+: high IHH levels.

IHH−: low IHH levels.
